# Comparative Analysis of Selected Physicochemical Properties of Pozzolan Portland and MTA-Based Cements

**DOI:** 10.1155/2014/831908

**Published:** 2014-08-12

**Authors:** Maura Cristiane Gonçales Orçati Dorileo, Ricardo Dalla Villa, Orlando Aguirre Guedes, Andreza Maria Fábio Aranha, Alex Semenoff-Segundo, Matheus Coelho Bandeca, Alvaro Henrique Borges

**Affiliations:** ^1^Faculty of Dentistry, University of Cuiabá, Avenida Manoel José de Arruda 3.100, Jardim Europa, 78065-900 Cuiabá, MT, Brazil; ^2^Master Program in Chemistry, Mato Grosso Federal University, Avenida Fernando Corrêa da Costa 2367, Boa Esperança, 78060-900 Cuiabá, MT, Brazil; ^3^Master Program in Dentistry, UNICEUMA, Rua Josué Montello 01, Renascença, 65075-120 São Luís, MA, Brazil

## Abstract

Physicochemical properties of pozzolan Portland cement were compared to ProRoot MTA and MTA BIO. To test the pH, the samples were immersed in distilled water for different periods of time. After the pH analysis, the sample was retained in the plastic recipient, and the electrical conductivity of the solution was measured. The solubility and radiopacity properties were evaluated according to specification 57 of the American National Standard Institute/American Dental Association (ANSI/ADA). The statistical analyses were performed using ANOVA and Tukey's test at a 5% level of significance. Pozzolan Portland cement exhibited pH and electrical conductivity mean values similar to those of the MTA-based cements. The solubilities of all tested materials were in accordance with the ANSI/ADA standards. Only the MTA-based cements met the ANSI/ADA recommendations for radiopacity. It might be concluded that the pH and electrical conductivity of pozzolan Portland cement are similar to and comparable to those of MTA-based cements.

## 1. Introduction

Vestiges of endodontic treatment failure, characterized by the presence of apical periodontitis and posttreatment symptomatology, are important indicators in which further intervention is required [1]. In clinical situations in which it is not possible to correct the condition by orthograde access, a surgical root canal treatment to save the teeth is required [2]. An apicoectomy with retrograde filling is an apical surgery with root resection followed by a class I cavity confection and placement of a retrograde material [3–5]. In the 1990s, to overcome the limitations of the retrofilling materials, mineral trioxide aggregate (MTA) was developed [6–8]. MTA is a powder containing fine hydrophilic particles of tricalcium silicate, tricalcium aluminate, tricalcium oxide, and silicate oxide that harden when in contact with water [9, 10]. Some authors have described MTA as a composition of ordinary Portland cement (PC), a material frequently used in civil engineering applications, with the addition of bismuth oxide for radiopacity [7]. PC and MTA display similar antimicrobial activity, biocompatibility, sealing ability, marginal adaptation, tissue and periradicular healing, dentin barrier formation, dimensional stability, and moisture tolerance [8].

Various studies have focused on modifying the current MTA formulation or developing a new material based on PC that could overcome the handling characteristics of MTA [11–17]. Kogan et al. [13] evaluated the effects of various additives on the setting properties of MTA and observed a decreased setting time and superior handling properties when NaOCl was added to the mixture. Wiltbank et al. [15] added classic PC accelerators (calcium chloride, calcium nitrite/nitrate, and calcium formate) to gray and white MTA and PC and observed that the additives significantly accelerated the setting reaction of the tested materials. Despite these favorable results, it has been reported that the use of additives could negatively interfere with some physicochemical and biological properties of MTA [16].

The numerous types of PC in Brazil are classified according to their composition [18]. Pozzolan PC is a mixture of PC and pozzolan, which, if dispersed in water and kept under certain conditions, eventually produces solutions unsaturated with calcium hydroxide [19]. The pozzolan material reduces the permeability and ionic diffusibility, increases the stability and durability, improves the performance compared to the action of the sulfates/alkali-aggregate reaction, reduces the hydration heat, and increases the compressive strength of the cement [18, 19]. According to the American Concrete Institute [20], pozzolan is a siliceous or siliceous and aluminous material that possesses little or no cementitious value in itself; however, in a finely divided form and in the presence of water, it chemically reacts with calcium hydroxide at ordinary temperatures to form compounds possessing cementitious properties. Recently, a more rapidly setting MTA-based cement was developed using small particles of pozzolan cement without chemical accelerators that presented biocompatibility and osteogenicity properties similar to those of ProRoot MTA [21, 22]. MTA-based pozzolan cement demonstrated a much shorter setting time than ProRoot MTA as well as antiwashout characteristics and a lack of surrounding marginal gaps [21].

Ørstavik [23] reported that, before the clinical use of a new material, it is necessary to perform several standardized* in vivo* and* in vitro* tests to confirm the physicochemical and biologic properties and the effect of the novel material on human. Although some physical properties and biocompatibility of MTA-based pozzolan cement are documented [20, 21], no study has directly compared selected physical and chemical properties of MTA-based cements and pozzolan PC. To introduce other possibilities for the development of a novel retrofilling material, the purpose of this study was to evaluate the solubility, hydrogenic potential, electrical conductivity, and radiopacity of pozzolan PC in comparison to those characteristics of ProRoot MTA and MTA BIO.

## 2. Material and Methods

### 2.1. Tested Materials

The materials used in this study were distributed in the following groups: ProRoot MTA (Dentsply, Tulsa Dental Products, Tulsa, OK, USA), MTA BIO (Angelus Soluções Odontológicas, Londrina, PR, Brazil), and pozzolan Portland cement (Votorantim, São Paulo, SP, Brazil). The MTA-based cements were mixed according to the manufacturers' recommendations. The pozzolan PC was mixed with distilled water at a water-to-powder ratio of 1 : 3. Temperature control (23 ± 2°C) and relative humidity were maintained. The chemical composition for the materials, according to the manufacturers, is described in Table 1.

The solubility and radiopacity, after setting for all the tested materials, were measured, according to the ANSI/ADA [24] specification 57 for root canal sealing materials and as suggested by Carvalho-Junior et al. [25].

### 2.2. Solubility

Five samples (1.5 mm thickness and 7.75 mm inner diameter) were used for each material. The cement was prepared and inserted into the mold. In sequence, a 0.5 mm diameter waterproof nylon was inserted in the softened cement. After duration of three times the setting time, the sample was removed from the mold and weighed on a precision scale of 0.0001 g (Ohaus Corporation, NJ, USA). The sample, suspended by the nylon, was placed in a wide-mouthed plastic recipient containing 7.5 mL of distilled water, avoiding contact with the internal wall. This container was hermetically closed and placed in an incubator at a constant temperature of 37 ± 2°C for 24 hours. After this time, the sample was removed and the excess water was removed with absorbent paper. The sample was maintained in a dehumidifier for 24 hours, after which it was weighed a second time. The solubility of the material was considered as the percentage of the lost mass compared to the initial mass. Five repetitions were considered for each material.

### 2.3. Hydrogenic Potential (pH)

Five samples (1.5 mm thickness and 7.75 mm inner diameter) were used for each material. Each cylinder was sealed in a flask containing 7.5 mL of distilled water. Distilled water pH measurements (PH30 Sensor Corning; Corning Inc., NY, USA) were taken with a pH meter at 1, 3, 5, 15, and 30 min; 1, 2, 3, 4, 6, 9, 12, 24, 48, and 72 h; and 4, 6, 7, 15, and 30 days after spatulation. During the experiment, the pH was analyzed for each sample in the same plastic recipient without liquid substitution. The pH was measured 5 times for each material. The mean values and standard deviations were recorded for all the measurements.

### 2.4. Electrical Conductivity

After the pH analysis, the sample was retained in the plastic recipient and the electrical conductivity of the solution was measured. All 5 samples of each material were analyzed with a conductivimeter (Marconi CA-150, Piracicaba, SP, Brazil). The device was calibrated according to a calibration curve obtained from a solution of 1.412 *μ*S/cm.

### 2.5. Radiopacity Test

Five acrylic plates (2.2 cm × 4.5 cm × 1 mm) with 3 holes measuring 1 mm in depth and 5 mm in the internal diameter were fabricated. The acrylic plates were placed onto a glass plate covered by cellophane paper, and each orifice was filled with one of the tested cements. For the radiographic exposure, each acrylic plate containing a cement sample was positioned with another acrylic plate (1.3 cm × 4.5 cm × 1 mm), which contained a graduated aluminum stepwedge varying from 1 to 10 mm in thickness and uniform steps of 1 mm each. The set of plates was built with standardized measurements in a manner by which they would correspond exactly to the sensor size (phosphor plate) and was obtained from DigoraTM system (Soredex, Orion Corporation, Helsinki, Finland) and used for the data collection. A 70 kVp and 8 mA radiograph machine, Spectro 70X (Dabi Atlante, Dabi Atlante Indústrias Médico Odontológicas Ltda, Ribeirão Preto, SP, Brazil), was used. The focus-object distance was 30 cm (ANSI/ADA, 2000), and the exposure time was 0.2 s, as instructed for the digital radiography of phosphor plates by the manufacturer. An acrylic-positioning device with metallic fastener-held sensors provided an adequate and standardized focus-object distance. The radiograph machine head was fixed on the same position with a central beam presenting a 90° angle of incidence with the acrylic/sensor surface plates set. A rectangular collimator (Dabi Atlante, Dabi Atlante Indústrias Médico Odontológicas Ltda), presenting a 3 × 4 cm aperture, reduced possible secondary radiation by being attached to the end of cylinder. The sensor, after being exposed, was inserted into the laser optical reader of Digora for Windows 5.1 software. As soon as the first image was revealed on screen, the parameters suggested by the system were established, allowing image standardization. The same phosphor plate was used for all the exposures to avoid possible differences between the plates. The system performed a radiographic density reading over the images of each cement revealed on the screen and of the steps on an aluminum stepwedge, resulting in a numeric value for each reading. This value was recorded by the evaluator. After evaluating the 5 acrylic sets of plates, 5 measurements for each type of cement and for each step of the aluminum scale were obtained. The mean values of the radiographic density and graduated aluminum stepwedge were determined for each material. The mean values were obtained by a single evaluator previously trained and blinded with regard to the different groups.

### 2.6. Statistical Analysis

Statistical analyses were performed for the solubility, pH, electrical conductivity, and radiopacity using ANOVA and Tukey's test at the 5% level of significance. When the sample distribution was nonnormal, nonparametric analyses of variance were performed with a Kruskal-Wallis test (*α* = 0.05). All the statistics and probabilistic errors were calculated with IBM SPSS 21.0 software (SPSS Inc., Chicago, IL, USA).

## 3. Results

The mean, standard deviation, and significant differences in the physiochemical properties (solubility, pH, electrical conductivity, and radiopacity) of the tested materials are shown in Table 2.

### 3.1. Solubility

According to ANSI/ADA specification 57 [22] a root canal sealer should not exceed 3% of the mass when the solubility of the set material is tested. The results showed agreement with the ANSI/ADA requirements. However, significant differences were observed between the tested materials, with MTA-based cements (ProRoot MTA and MTA BIO) presenting the lowest values of solubility (*P* < 0.05) (Table 2).

### 3.2. pH Analysis

**Figure 1:**
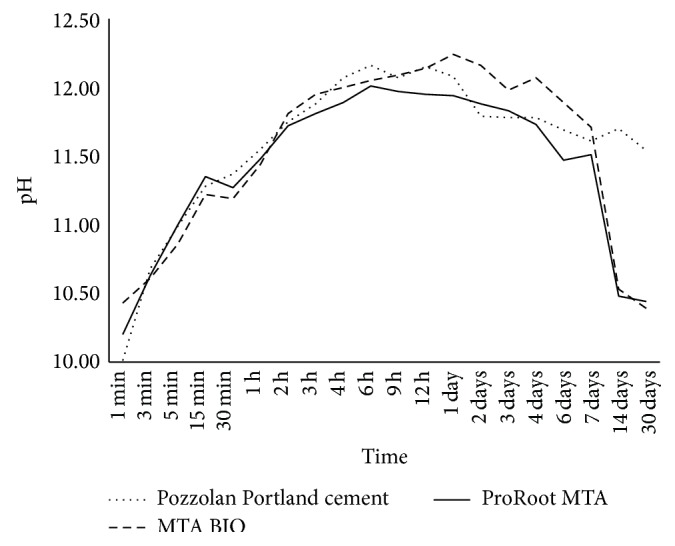
Hydrogenic potential changes in the tested materials according to different periods of time. No significant difference was observed in the mean values for the pH reading of each tested material (*P* > 0.05). During the experimental period, the values of pH were high (alkaline) from the beginning until the end.

The change in pH as a function of time is shown in Figure 1. The pH values for the cements ranged from 10.01 to 12.24. At immersion for 1 min, significant differences were observed with other time periods (*P* < 0.05). No significant difference was observed in the mean values for the pH reading of each tested material (*P* > 0.05) (Table 2).

### 3.3. Electrical Conductivity

**Figure 2:**
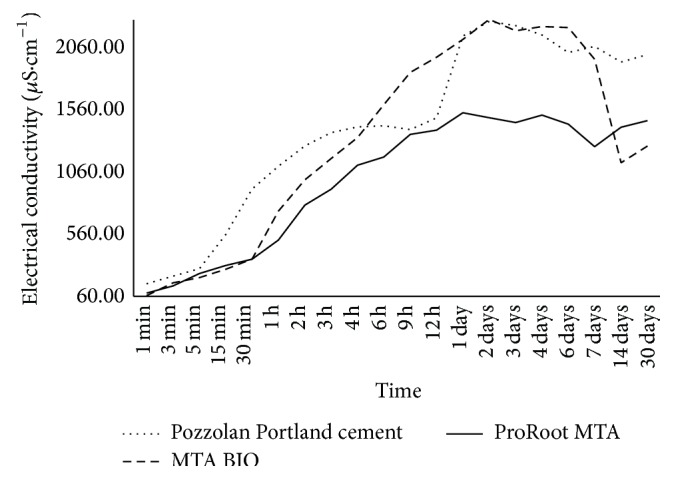
Electrical conductivity (*μ*S/cm) evaluation according to different time periods. At 1 min and 1, 2, 3, 4, and 6 days, a significant difference in electrical conductivity was observed (*P* < 0.05).

The results indicated that the conductivity of the materials was not significantly different (*P* > 0.05). At 1 min and 1, 2, 3, 4, and 6 days, a significant difference in conductivity was observed (*P* < 0.05). Alternatively, at other periods of time, differences were not observed between the samples (*P* > 0.05) (Figure 2).

### 3.4. Radiopacity

ProRoot MTA presented the highest radiopacity mean values among the tested materials (177.40 ± 7.30 mm Al), followed by MTA BIO (165.80 ± 3.27 mm Al). Both materials overcame 3 steps from the aluminum stepwedge, which is the minimum recommended by ANSI/ADA specification 57 [24], whereas the pozzolan Portland cements did not meet this requirement (109.40 ± 3.50 mm Al). The statistical analysis demonstrated a difference between the tested materials (*P* < 0.05) (Table 2).

## 4. Discussion

Because of the lack of specific standards for testing the physical properties of retrofilling materials, published studies have followed ANSI/ADA specification number 57 for endodontic sealing materials [17, 26] and the ISO 6876 specification for zinc oxide and eugenol endodontic sealing materials [4] to support and reference studies analyzing the physicochemical properties of MTA and PC. Under clinical conditions, retrofilling and root filling materials remain in close contact with the periodontal tissues; thus, the ANSI/ADA standard was assumed to be applicable to the materials under investigation [26], following the modifications proposed by Carvalho-Junior et al. [25], which allow the reduction of 80% in the volume of the material for conducting tests without involvement or interference in the results.

During the last decades, endodontic research has been characterized by a constant search for a retrograde filling material superior to MTA in working characteristics that provides simple dental management and promotes healing and cellular regeneration [14]. PC has been extensively investigated as a viable alternative for MTA in endodontic applications [27], and although MTA-based pozzolan cement has been developed as a dental material [21, 22], limited information regarding pozzolan PC has been published. In this study, the solubility, pH, electrical conductivity, and radiopacity of pozzolan PC were analyzed and compared to those characteristics of MTA-based cements to discover a new retrofilling material that is equally effective as MTA. Understanding the physical and mechanical properties of a material is critical to determining its suitability for clinical use as a restorative material as well as to dictate its clinical applications [12].

In the solubility analysis, all the tested materials met the ANSI/ADA specification 57 requirements [24], according to which a root canal sealer should not exceed 3% of the initial mass when the solubility of the set material is tested. Pozzolan PC was found to present significantly greater solubility than ProRoot MTA and MTA BIO. This result is in accordance with previous reports [4, 28] and is explained by the chemical surface composition of these materials, which present different structures after the setting time reaction [26, 29]. Dammaschke et al. [30] observed that MTA-based cements present less concentrated levels of sulfur and potassium and increase calcium content at the surface of the material, whereas PC presents a higher sulfur content, which is related to a greater amount of gypsum. The higher gypsum content in PC has been suggested as one reason for the increased solubility [26]. The addition of bismuth oxide, which is insoluble in water, to MTA-based cements [31] is an additional cause of MTA insolubility [9]. Islam et al. [12] found contradictory results, with white ProRoot MTA presenting significantly greater solubility than PC. Bodanezi et al. [32] evaluated the immediate and delayed solubility of gray MTA-Angelus and PC; the authors observed that the residual mass separated from MTA-Angelus was significantly higher and, consequently, it was more soluble when completely immersed in an aqueous environment throughout 672 hours [32]. Vivan et al. [33] evaluated the solubility of two commercially MTA-based cements (MTA-Angelus and MTA BIO) and of three experimental cements (light-cured MTA, PC with 20% bismuth oxide and 5% calcium sulfate, and an epoxy resin-based cement); the materials that showed the lowest solubility values were the epoxy resin-based cement, PC with bismuth oxide, and light-cured MTA. The highest solubility values were shown in white MTA-Angelus and MTA BIO. These distinct results might be attributed to the different methodologies used in the studies, including the time of immersion of the material, the type of MTA, the type of PC, and the powder-to-liquid ratio [9, 12]. Solubility testing standards recommend immersion of the materials only after setting is complete, which is impossible to achieve under clinical conditions because the materials are immediately in contact with oral fluids [9, 16, 17, 32].

All of the analyzed cements are rich in calcium oxide, which converted to calcium hydroxide upon contact with water [34]. This process causes pH increase through the dissociation of calcium and hydroxyl ions [35]. The setting reaction of the cement is based on the relationship of the anhydrous cement compounds with water [2, 10, 13, 15, 16]. In this reaction, the individual components of the cement are attacked and react together to form hydrated compounds [29]. The hydration is basically a silicate hydrolysis, releasing lime that separates in the form of calcium hydroxide [34, 36] and a calcium silicate hydrate, producing less basicity [9]. For this reason, after the setting time, these cements are considered as calcium hydroxide in a silicate matrix [2, 29]. The presence of calcium hydroxide is responsible for the high alkalinity of the medium [10, 29], which is crucial for clinical reasons and related to the ability of MTA and PC to promote healing [22, 34]. The immediate increase in pH after the material immersion is caused by the reaction that occurs when cement comes in contact with water, resulting in a saturated calcium hydroxide solution [12]. During the experimental period, the pH values remained high (alkaline) from the beginning until the end. The method for pH measurement in this study was extensively described [15, 16, 28, 36] and did not suggest a distilled water change after each period of analysis. Hungaro Duarte et al. [35] and Vivan et al. [33] changed the water for each pH measurement performed; thus, after each reading, the pH of the solution returned to a value close to that of distilled water, requiring a long time to reach a higher value. The pH values observed by Duarte et al. [35] and Vivan et al. [33] were lower than those observed in this study.

Electrical conductivity is a natural facility by which each material conducts its specific electric charge [37]. This capacity is related to the quantity of ions released to the medium and is directly proportional to the solubility of the material [37]. The results of this study indicated that the concentration of ions in solution increased as the solubility of the sample increased, which led to higher conductivity values during the test period. This behavior was observed in all of the cements. The sample components solubilize at different rates and possess different solubility products [38]. Considering the complexity of the materials, the ionic equilibrium is equally complex. Calcium is the main element present in these cements and should be considered the common-ion effect. The conductivity values of the cements were similar, suggesting that all the samples were affected in the similar mechanism by the solvent. Although the conductivity significantly increased over time, the electrical conductivity should eventually stabilize because of solution saturation [37].

Retrofilling materials should present adequate radiopacity to be distinguished from the surrounding anatomical structures such as teeth and alveolar bone and to reveal empty spaces and inappropriate contours [27]. The radiopacity of ProRoot MTA and MTA BIO are adequate, according to the ANSI/ADA requirements [24], which specify that an endodontic sealing material should present radiopacity correspondent to at least 3 mm Al. This finding was expected because these materials contain bismuth oxide, which is added to improve the radiopacity characteristic of the material [30, 31, 35]. According to our results, ProRoot MTA was significantly more radiopaque than MTA BIO. This finding is in accordance with previous studies [12, 26] and is explained by the difference in the chemical composition of these materials. Song et al. [31], using X-ray diffraction assays, showed that ProRoot MTA had a higher content of bismuth oxide than MTA-Angelus. Pozzolan PC exhibited the lowest radiopacity mean value, in accordance with literature that reported that PC materials have intrinsic radiopacity values lower than 3 mm Al [12, 39], the minimum condition recommended by the ANSI/ADA [24]. This is a major drawback of PC if it is to be used clinically. To address this issue, PC was associated with different radiopacifiers aiming to promote satisfactory radiopacity higher than dentin [27, 39, 40]. Further investigations are required to elucidate cement/radiopacifier agent mixture interference with the physicochemical properties and biocompatibility of MTA-based and Portland cements [36].

## 5. Conclusions

Considering the present results and the inherent limitations of the methodology used, we might conclude that the solubilities of the tested materials were in accordance with the ANSI/ADA standards. Only the MTA-based cements met the ANSI/ADA recommendations referring to radiopacity. It might be concluded that in pH and electrical conductivity, pozzolan PC was similar to and comparable to the MTA-based cements.

## Figures and Tables

**Table 1:** Composition of the tested materials and manufacturer.

Cement	Composition	Manufacturer
Pozzolan PC	Clinker and gypsum (84–45%), pozzolan material (15–50%), and carbonate material (0–5%)	Votorantim Cimentos, São Paulo, Brazil
MTA BIO	Portland cement (80%) and bismuth oxide (20%)	Ângelus Ind. Prod., Londrina, Brazil
ProRoot MTA	Portland cement (75%), bismuth oxide (20%), and gypsum (5%)	Dentsply, Tulsa Dental, Tulsa, USA

**Table 2:** Mean, standard deviation, and statistic comparison of physicochemical properties for each tested material.

Physicochemical properties	Power (%)	Tested materials		
		Pozzolan PC	MTA BIO	ProRoot MTA
Solubility (%)	89	0.52 ± 0.8^a^	0.06 ± 0.04^b^	0.05 ± 0.03^b^
pH	57	11.44 ± 0.59^a^	11.53 ± 0.64^a^	11.42 ± 0.57^a^
Electrical conductivity (*μ*S/cm)	39	1472.69 ± 651.49^a^	1291.8 ± 778.51^a^	987.59 ± 521.94^a^
Radiopacity (mm Al)	98	109.40 ± 3.50^a^	165.80 ± 3.27^b^	177.40 ± 7.30^c^

^*^The same superscript letters represent no statistically significant difference (*P* < 0.05).
